# Synthesis of indolo[3,2-*b*]carbazole-based new colorimetric receptor for anions: A unique color change for fluoride ions

**DOI:** 10.3762/bjoc.6.12

**Published:** 2010-02-08

**Authors:** Ajit Kumar Mahapatra, Giridhari Hazra, Prithidipa Sahoo

**Affiliations:** 1 Department of Chemistry, Bengal Engineering and Science University, Shibpur, Howrah 711103, India, Tel.: +91 33 2668 4561; Fax: +91 33 2668 4564

**Keywords:** anion binding, colorimetry, fluorescence quenching, fluoride binding, indolocarbazole

## Abstract

A novel indolocarbazole-based chemosensor **1** containing hydrogen bond donor moieties has been established as a selective colorimetric and fluorometric sensor for F^−^ in CH_3_CN/H_2_O (4:1 v/v). Upon the addition of a series of tetrabutylammonium salts to receptor **1** in aqueous CH_3_CN, only when the counter ion was F^−^ was a significant color change (from light violet to dark orange) observed.

## Introduction

The design and synthesis of chromogenic receptors for biologically important and environmentally harmful anion pollutants has attracted considerable attention in supramolecular chemistry [1–2]. Most of the synthetic chemosensors generally involve covalent linking of an optical-signaling chromophoric fragment to a neutral anion receptor containing urea [3], thiourea [4], amide [5], phenol [6–7], or pyrrole [8] subunits, which can provide one or more H-bond donor sites for selective binding and sensing of certain anions, especially F^−^, AcO^−^, H_2_PO_4_^−^, etc. In particular, the selective sensing of fluoride has gained attention due to its significant role in clinical treatments e.g. dental care [9], osteoporosis [10] and for the detection of fluoride in bones as a result of over-accumulation [11]. This diversity of function, both beneficial and otherwise, makes the problem of fluoride ion detection of considerable interest. In this context, a colorimetric chemosensor is of particular interest due to its simplicity. Color changes that can be detected by the naked eye are widely used as signals for detection of anions without the need for any expensive equipment or even without the requirement of any equipment whatsoever [12–13].

**Figure 1:**
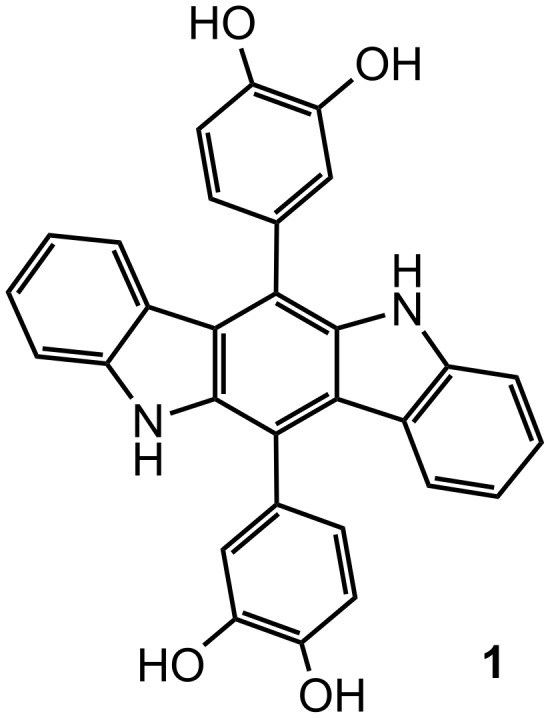
The structure of the indolocarbazole-based chemosensor **1**.

In the last few years, although some synthetic receptors have become available for fluoride ions [14–28], there is a paucity of reports on selective naked-eye chemosensors for fluoride [29–31]. Nitrophenyl, nitronaphthalene urea [32–34], naphthalene triphenyl-phosphonium [35], benzimidazolyl pyridine [36–37] and oxidized bis(indolyl)methane [38] as signal units for fluoride have been reported as chromogenic chemosensors, but a indolocarbazole ligand for the anion remains to be developed. Recently, Bhardwaj et al. reported a tripodal receptor [39] bearing catechol groups [40] for the chromogenic sensing of fluoride ions. Numerous bis(indolyl)methanes and their derivatives exhibit important biological activities [41]. Therefore, there has been great interest in the synthesis of bisindole compounds both naturally occurring and totally synthetic. As an extension of our work [42] on supramolecular chemistry, we now report a simple and new indolocarbazole-based molecular receptor **1** for the selective sensing of anions by investigating the effect of the addition of tetrabutylammonium salts ([Bu_4_N]^+^X^−^, X = F^−^, Cl^−^, Br^−^, I^−^, AcO^−^, HSO_4_^−^, and H_2_PO_4_^−^). Receptor **1** (Figure 1) was particularly important as a chemosensor for fluoride owing to its noticeable color change in the presence of F^−^ ions.

## Results and Discussion

### Synthesis

**Scheme 1:**
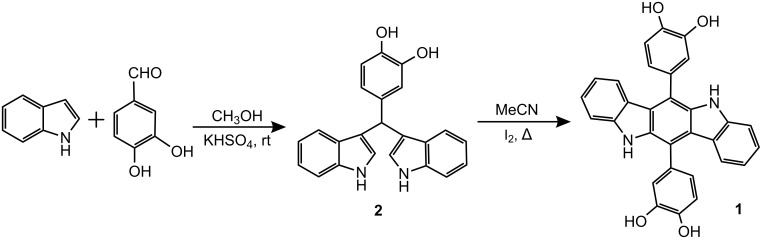
Synthesis of receptor **1**.

Receptor **1** was synthesized [43–44] according to Scheme 1. Condensation of indole with 3,4-dihydroxybenzaldehyde by the reported procedure yielded intermediate **2**, which was found to be unstable at room temperature. Subsequent heating of **2** in CH_3_CN in the presence of I_2_ for 45 min afforded the desired receptor **1** in 82% yield.

**Figure 2:**
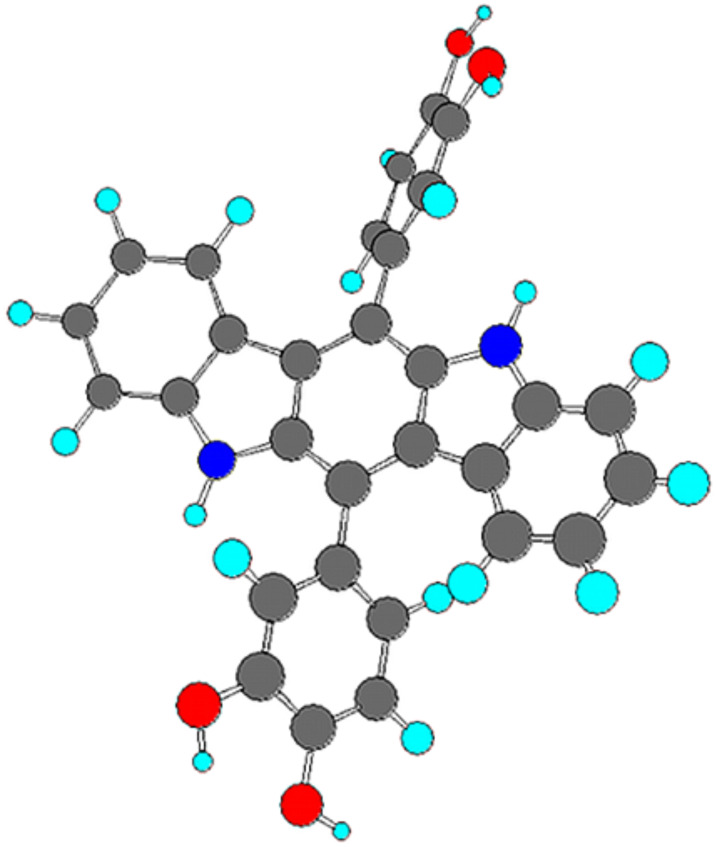
The AM1 optimized structure of receptor **1** (heat of formation = −8.29 kcal/mol).

To look into the orientation of hydrogen bond donors around the carbazole motif, we optimized the structure by the AM1 method [45] (Figure 2). It is evident from Figure 2 that the two catechol units do not lie in the same plane as the carbazole unit.

### Interaction studies

#### UV–vis study

The anion-binding properties of receptor **1** were investigated by UV–vis, fluorescence and ^1^H NMR spectroscopic methods. The sensing ability of chemosensor **1** with a series of tetrabutylammonium salts ([Bu_4_N]^+^X^−^, X = F^−^, Cl^−^, Br^−^, I^−^, AcO^−^, HSO_4_^−^, and H_2_PO_4_^−^) in CH_3_CN/H_2_O (4:1 v/v) was monitored by UV–vis absorption studies and by ‘naked-eye’ observation. The tetrabutylammonium salt (TBAX) under investigation was added to a solution of receptor **1** (*c* = 1.1 × 10^−4^ M) in the above noted solvent mixture.

**Figure 3:**
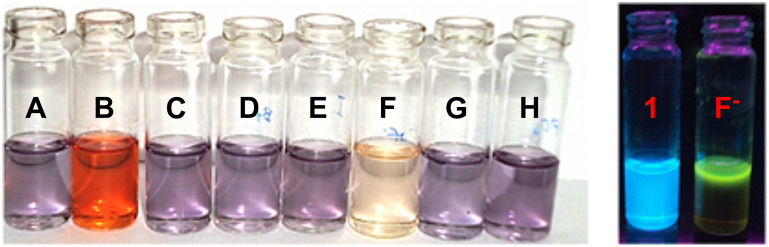
Color changes of receptor **1** (A) (*c* = 1.1 × 10^−4^ M) in CH_3_CN/H_2_O (4:1 v/v) on addition of tetrabutylammonium salt (TBAX), X = F^−^ (B), Cl^−^ (C), Br^−^ (D), I^−^ (E), AcO^−^ (F), HSO_4_^−^ (G), and H_2_PO_4_^−^ (H) (left side); green fluorescence observed on addition of F^−^ ion to receptor **1** (right side).

In the naked-eye experiments, receptor **1** (*c* = 1.1 × 10^−4^ M) in CH_3_CN/H_2_O (4:1 v/v) showed distinct color changes from light violet to dark orange and pale pink, respectively, in the presence of two equivalent amounts of TBAF and TBAOAc (Figure 3). In the fluorescence study, the sky blue color of **1** changed to a green color on the addition of TBAF. Importantly, the receptor was found to be insensitive to the addition of large excess of Cl^−^, Br^−^, I^−^, HSO_4_^−^, and H_2_PO_4_^−^ (even up to 100 equiv). The change in color was due to the deprotonation of phenolic OH groups followed by hydrogen bonding with fluoride ions. The strong hydrogen bonding to, or deprotonation/protonation of, the indolocarbazole moiety might modulate the electronic properties of chromophore [46] and give rise to significant color changes.

**Figure 4:**
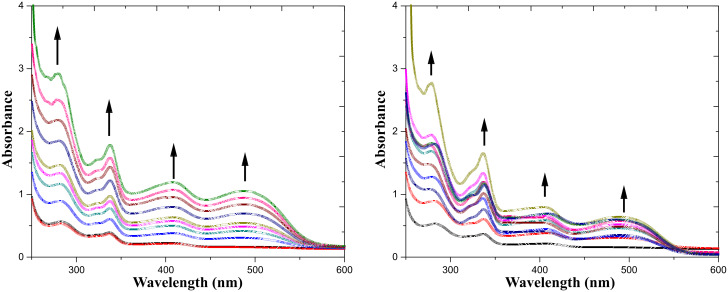
UV spectral change of receptor **1** (*c* = 1.1 × 10^−4^ M) upon gradual addition of [Bu_4_N]^+^F^−^ (left side) and [Bu_4_N]^+^AcO^−^ (right side) in CH_3_CN/H_2_O (4:1 v/v) (*c* = 1.1 × 10^−4^ M).

The interaction of receptor **1** (*c* = 1.1 × 10^−4^ M) with F^−^ was investigated in aqueous CH_3_CN solvent in more detail by UV–vis spectroscopic titration (Figure 4). Receptor **1** itself displays two absorption bands at 283 and 338 nm in CH_3_CN/H_2_O (4:1 v/v). Upon the gradual addition of F^−^, the absorbance increases by different extents. On increasing the concentration of F^−^, two new absorption bands appear at 408 and 491 nm, with the effect that the solution instantaneously changes color from light violet to dark orange. These two new bands can be ascribed to the deprotonated receptor. Figure 4 shows the F^−^-induced UV–vis spectral change of receptor **1** at different concentrations of fluoride ion in CH_3_CN/H_2_O (4:1 v/v) (left side). A similar, but less remarkable spectral change, was observed upon addition of AcO^−^ (right side) where a color change from light violet to light pink was achieved upon the addition of 10 equiv of AcO^−^.

**Scheme 2:**
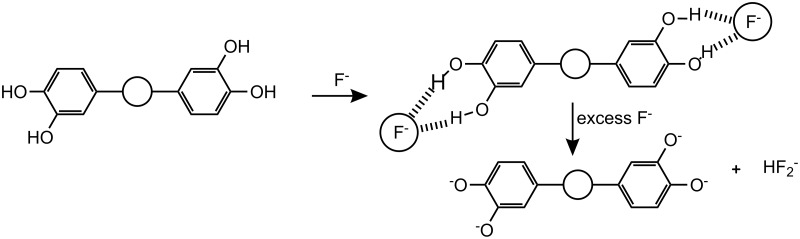
Schematic representation (the circles represent the indolocarbazole moiety) of the two-step process leading to receptor deprotonation with basic fluoride anions.

**Figure 5:**
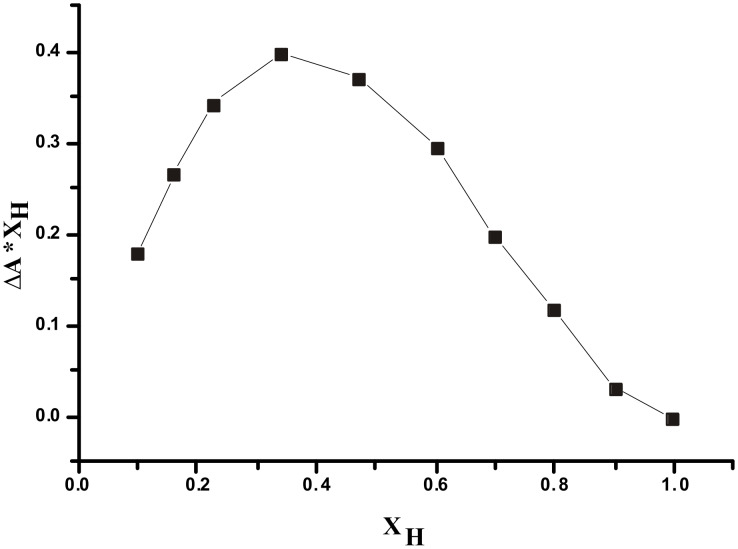
The Job plot of **1** with fluoride ion from UV method in CH_3_CN/H_2_O (4:1 v/v).

The spectral behavior indicated that deprotonation of the phenolic OH as well as NH groups by F^−^ (Scheme 2), and not hydrogen bonding to it, is responsible for the drastic color change [47], as a result of a change in the optical properties of chromogenic indolocarbazole skeleton. This is in agreement with the NMR titration data. Such deprotonation was related to the acidity of the H-bond donor site and the particular stability of the [HF_2_]^−^ ion. The stoichiometry of **1** with F^−^ was determined to be 1:2 from the Job plot [48] (as shown in Figure 5).

Parallel investigations were carried out with a series of other anions (Cl^−^, Br^−^, I^−^, AcO^−^, HSO_4_^−^, and H_2_PO_4_^−^). Similar phenomena with negligible perturbations of UV–vis absorption were observed with no noticeable change in color in the cases of Cl^−^, Br^−^, I^−^, HSO_4_^−^, and H_2_PO_4_^−^, even at levels of up to 100 equiv. Similar anion-sensing properties were also observed in the more polar solvent DMSO (all UV–vis spectra in supporting information).

Deprotonation of receptor **1** also took place with the basic anion AcO^−^ and the development of a light pink color was observed after the addition of excess anion. No deprotonation took place in the presence of less basic anions such as Cl^−^, Br^−^, I^−^, HSO_4_^−^, and H_2_PO_4_^−^. The receptor **1** is able to bind fluoride ion more strongly than other anions, since the catechol moiety is particularly effective in binding smaller anions. The deprotonation occurred at a slightly higher concentration of acetate than fluoride due to higher electronegativity, smaller size, and higher basicity of F^−^ ions, which make them bind strongly with receptor **1** [49]. The binding constants (*K*_a_) of receptor **1** (Table 1) with fluoride and other ions were determined by considering a hydrogen-bonded complex with the first two equivalents of anions in a 1:2 ratio of receptor and anion complex, and that, subsequently, the second equivalent of anion (addition of excess F^−^ ion) abstracts a HF fragment to give [HF_2_]^−^.

**Table 1:** Association constants^a^ of receptor **1** (R**1**) with [Bu_4_N]^+^X^−^ salts (X = F^−^, Cl^−^, Br^−^, I^−^, AcO^−^, HSO_4_^−^, and H_2_PO_4_^−^) in CH_3_CN/H_2_O (4:1 v/v) determined by UV–vis and fluorescence methods.

Guests	R1 (*K*_a_ M^−1^)	
	UV–vis method	Fluorescence method

F^−^	3.62 × 10^4^	8.21 × 10^4^
Cl^−^	7.92 × 10^3^	3.62 × 10^3^
Br^−^	4.29 × 10^3^	2.47 × 10^3^
I^−^	4.13 × 10^3^	2.27 × 10^3^
AcO^−^	1.21 × 10^4^	1.04 × 10^4^
HSO_4_^−^	1.14 × 10^3^	2.25 × 10^3^
H_2_PO_4_^−^	1.32 × 10^3^	6.65 × 10^3^

^a^All errors are ±0.8%.

A higher association constant was observed for fluoride ion than for other ions due to its strong hydrogen-bonding ability, small size, and better selectivity which resulted in a strong binding with receptor **1** [50].

#### Fluorescence study

**Figure 6:**
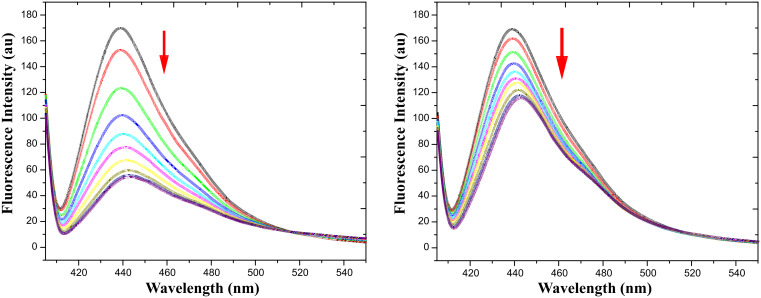
Fluorescence change of receptor **1** (*c* = 4.475 × 10^−5^ M) upon gradual addition of [Bu_4_N]^+^F^−^ (left side) and [Bu_4_N]^+^AcO^−^ (right side) in CH_3_CN/H_2_O (4:1 v/v) (*c* = 4.475 × 10^−5^ M) (λ_max_ = 443 nm).

**Figure 7:**
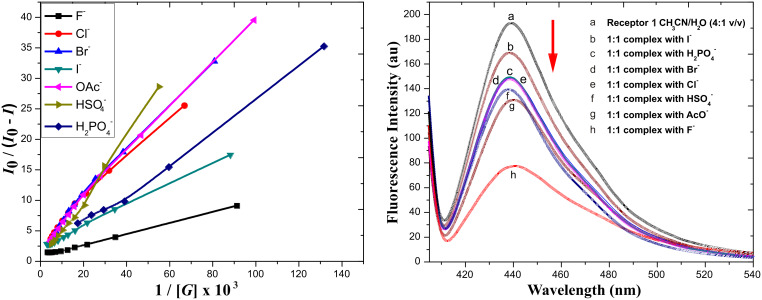
Binding constant calculation curves for receptor **1** vs F^−^, Cl^−^, Br^−^, I^−^, AcO^−^, HSO_4_^−^, and H_2_PO_4_^−^ (left side); fluorescence spectra of **1** after addition of 1:1 equivalent of receptor and anions (F^−^, Cl^−^, Br^−^, I^−^, AcO^−^, HSO_4_^−^, and H_2_PO_4_^−^) (right side).

Fluorescence spectroscopy studies were also carried out in order to evaluate the ability of **1** as a fluorescent anion sensor. Significant quenching of the fluorescence of **1** was observed upon addition of F^−^ ions to the solution of **1** (Figure 6, left side). In comparison, other anions, with the exception of AcO^−^ (Figure 6, right side), hardly altered the emission of **1**. A large quenching of intensity with respect to other anions (Figure 7, right side) was observed at 439 nm upon the addition of 2.0 equiv of [Bu_4_N]^+^F^−^. These results indicate that formation of hydrogen-bonded complex or deprotonation/protonation occurs by forming the anion of receptor **1**; the excited state was modified considerably leading to the quenching of fluorescence. A commonly accepted mechanism for the quenching phenomenon involves an inversion between the strongly emissive ππ^*^ and the poorly emissive nπ^*^ states of this fluorophore. Such a quenching results from a hydrogen bond interaction of phenolic OH with anions, which leads to the stabilization of the nπ^*^ state with respect to the ππ^*^ state and a subsequent decrease in the fluorescence emission intensity [51].

Analogous investigation of fluorescence was carried out with other [Bu_4_N]^+^X^−^ salts (X = Cl^−^, Br^−^, I^−^, HSO_4_^−^, and H_2_PO_4_^−^). In all cases, only slight quenching occurs on the gradual addition of the anions (Cl^−^, Br^−^, I^−^, HSO_4_^−^, and H_2_PO_4_^−^) to receptor **1**. The spectral variations observed for receptor **1** on titrating with different anions are given in the supplementary information.

#### ^1^H NMR study

**Figure 8:**
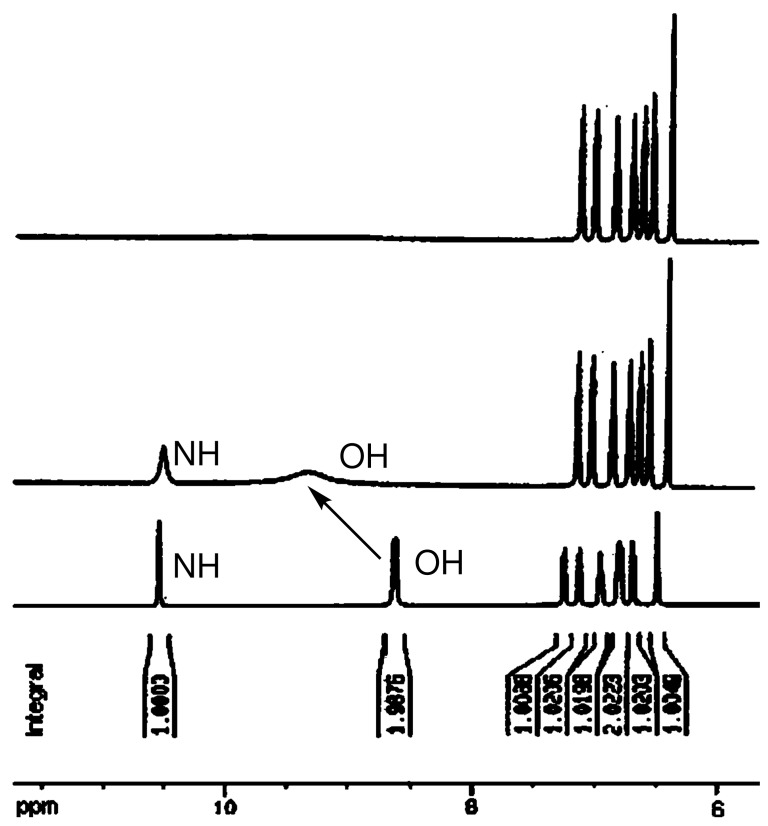
^1^H NMR spectra of receptor **1** (bottom), **1** with [Bu_4_N]^+^F^−^ 1:2 [receptor **1**:(Bu_4_N)^+^F^−^] (middle) and excess [Bu_4_N]^+^F^−^ (top).

The interaction of receptor **1** with F^−^ was corroborated by ^1^H NMR experiments carried out in DMSO-*d*_6_ (**1** has only limited solubility in CD_3_CN). A partial ^1^H NMR spectrum of receptor **1** is shown in Figure 8. It was found that the aromatic proton signals underwent upfield shifts with increasing F^−^ concentration. In the presence of equivalent amounts of [Bu_4_N]^+^F^−^, the signal for phenolic OH protons of **1** underwent large downfield shift (Δδ = 1.34 ppm) and the proton signal was broadened. These observations further indicated that the first added F^−^ establishes an H-bond interaction with the OH subunit of **1**, while an excess of F^−^ induces the deprotonation of the catechol moieties and NH proton, which brings electron density onto the π-conjugated framework through bond propagation, thus causing a shielding effect and inducing upfield shift of aromatic protons. The above mentioned results indicate that receptor **1** exhibits selective sensing for F^−^ (F^−^ > AcO^−^ >> other anions) in an appropriate solvent.

The fluoride- and acetate-induced deprotonation process is reversible as evidenced from the addition of CH_3_OH. The addition of the polar protic solvent methanol results in a gradual decrease of absorbance in UV–vis studies. This is presumably because the presence of a relatively high amount of protic solvent disfavors the formation of the deprotonated receptor. However, in the water–acetonitrile system, no color changes were observed upon addition of organic bases such as triethylamine whilst the addition of excess [Bu_4_N]^+^OH^−^ can deprotonate receptor **1** and induce a color change.

## Conclusion

In conclusion, a new colorimetric receptor **1** based on indolocarbazole was synthesized in high yield, which can form 1:2 complex with anions by multiple hydrogen-bonding interactions. Among the anions, only receptor **1** has higher selectivity for F^−^ and leads to a distinct color change that can be observed by the naked eye. The binding results with a series of anions suggest that receptor **1** efficiently binds F^−^ as established by UV–vis, fluorescence and ^1^H NMR spectroscopic methods. As a colorimetric anion sensor, the indolocarbazole-based receptor **1** displayed highly selective coloration for F^−^ ion even in the presence of other anions.

## Experimental

### General details

All reactions were carried out under a nitrogen atmosphere. Solvents were dried before use. Solvents for spectroscopic measurements were of spectroscopic or HPLC grade. The ^1^H NMR spectra were recorded on a Bruker AM-500 spectrometer. The ^1^H NMR chemical shift values are expressed in ppm (δ). UV–visible and fluorescence spectra measurements were performed on a JASCO V530 and a PerkinElmer LS-55 spectrofluorimeter, respectively. Receptor **1** and guest anions were dissolved in UV-grade acetonitrile and water (4:1 v/v). The corresponding absorbance values for receptor **1** were noted during titration and used for the determination of binding constant values. Binding constants were determined by using the expression *A*_0_/*A* − *A*_0_ = [ε_M_/(ε_M_ − ε_C_)](*K*_a_^−1^ *C*_g_^−1^ + 1), where ε_M_ and ε_C_ are molar extinction coefficients for receptor and the hydrogen-bonding complex, respectively, at selected wavelengths, *A*_0_ denotes the absorbance of the free receptors at the specific wavelength, and *C*_g_ is the concentration of [Bu_4_N]^+^X^−^ (X = F^−^, Cl^−^, Br^−^, I^−^, AcO^−^, HSO_4_^−^, and H_2_PO_4_^−^ anions). The measured absorbance *A*_0_/*A* − *A*_0_ as a function of the inverse of the guest anion concentration fits a linear relationship, indicating a 1:2 complexation of the receptor and anions. The ratio of the intercepts to the slope was used to determine the binding constant *K*_a_.

Geometric optimization of their stable conformation of receptor **1** at the AM1 level was carried out using the minimal valence basis as STO 3G in ArgusLab 4.0.1 software suite. We have refrained from citing calculated total energy value, the calculation being for molecule only in the gas phase.

### Receptor 1

3,3′-Bis(indolyl)-3,4-dihydroxyphenylmethane (**2**, 0.5 g, 1.41 mmol) in a round-bottom flask containing dry acetonitrile (5 mL), I_2_ (2 mol %) was added and the mixture refluxed for 45 min. The solid obtained was filtered, dried and recrystallized from a mixture of DMF–CHCl_3_. Yield 82%, mp 258 °C; ^1^H NMR (500 MHz, DMSO-*d*_6_): δ (ppm) 10.53 (s, 2H), 8.63 (bs, 4H), 7.24 (d, *J* = 8 Hz, 2H), 7.12 (d, *J* = 8 Hz, 2H), 6.94 (t, *J* = 7.5 Hz, 2H), 6.80 (t, *J* = 7.5 Hz, 2H), 6.68 (d, *J* = 8 Hz, 2H), 6.48 (d, *J* = 7.4 Hz, 2H), 6.43 (s, 2H); ^13^C NMR (125 MHz, DMSO-*d*_6_): δ (ppm) 145.1, 143.9, 137.1 (for two carbon), 135.1 (for two carbon), 125.9, 120.4, 119.4, 118.59, 118.0, 115.4, 115.1, 11.0, 110.1; FTIR (KBr, cm^−1^): 3472, 3430, 1521, 1457, 1262, 1224; C_30_H_20_N_2_O_4_ (473.1496); Anal. Calcd C, 76.26; H, 4.27; N, 5.93; O, 13.54; found C, 76.35; H, 4.19; N, 5.73; O, 13.60; HRMS (MH^+^ + 2): 475.21.

## Supporting Information

File 1 ^13^C NMR and mass spectra of the synthesized compound R**1** and its UV–vis and fluorescence spectra in the presence of different anions (Cl^−^, Br^−^, I^−^, HSO_4_^−^, and H_2_PO_4_^−^).
