# Supplementary material for: Synthesis of indolo[3,2-b]carbazole-based new colorimetric receptor for anions: A unique color change for fluoride ions
Source: Beilstein J Org Chem. 2010 Feb 8;6:12. doi: 10.3762/bjoc.6.12 (PMC2871001; doi:10.3762/bjoc.6.12)
Supplement: File 1 — 13C NMR and mass spectra of the synthesized compound R1 and its UV–vis and fluorescence spectra in the presence of different anions (Cl−, Br−, I−, HSO4−, and H2PO4−). [file Beilstein_J_Org_Chem-06-12-s001.pdf]

Supporting Information

Synthesis of indolo[3,2-*b*]carbazole-based new colorimetric receptor for anions: A unique color change for fluoride ions

Ajit Kumar Mahapatra*, Giridhari Hazra and Prithidipa Sahoo

Department of Chemistry, Bengal Engineering and Science University, Shibpur, Howrah – 711103, India.

*Corresponding author: Tel.: +91 33 2668 4561; fax: +91 33 26684564; e-mail: mahapatra574@gmail.com

Supporting Information File 1: ^{13}C NMR and mass spectra of the synthesised compound R**1** and its UV-vis and fluorescence spectra in the presence of different anions (Cl^-, Br^-, I^-, HSO_4^-, $H_2PO_4^-$).

Contents

Page

1. ^{13}C-NMR and HRMS Receptor **1** ……………………………………………………P2

2. UV–vis and Fluorescence spectra of receptor **1** with Cl^-, Br^-, I^-, HSO_4^-, $H_2PO_4^-$ [CH_3CN/H_2O (4:1 v/v)] and F^- (DMSO solvent) ………………………………P3-P5

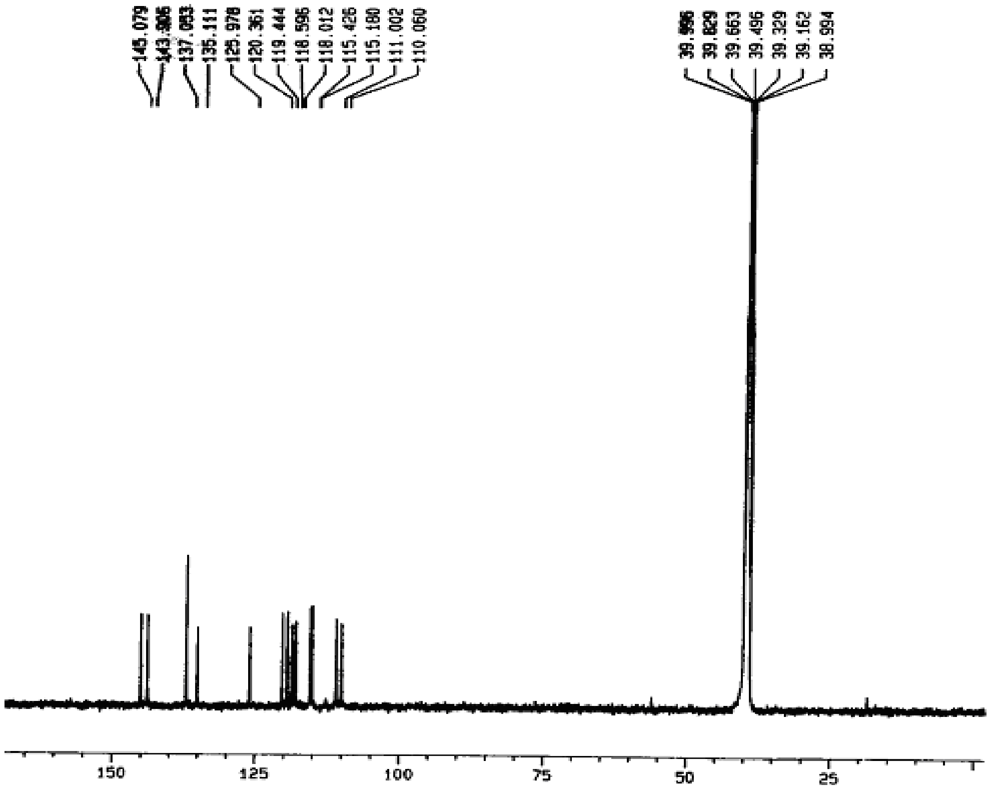

Figure 1: ^{13}C NMR of receptor **1.**

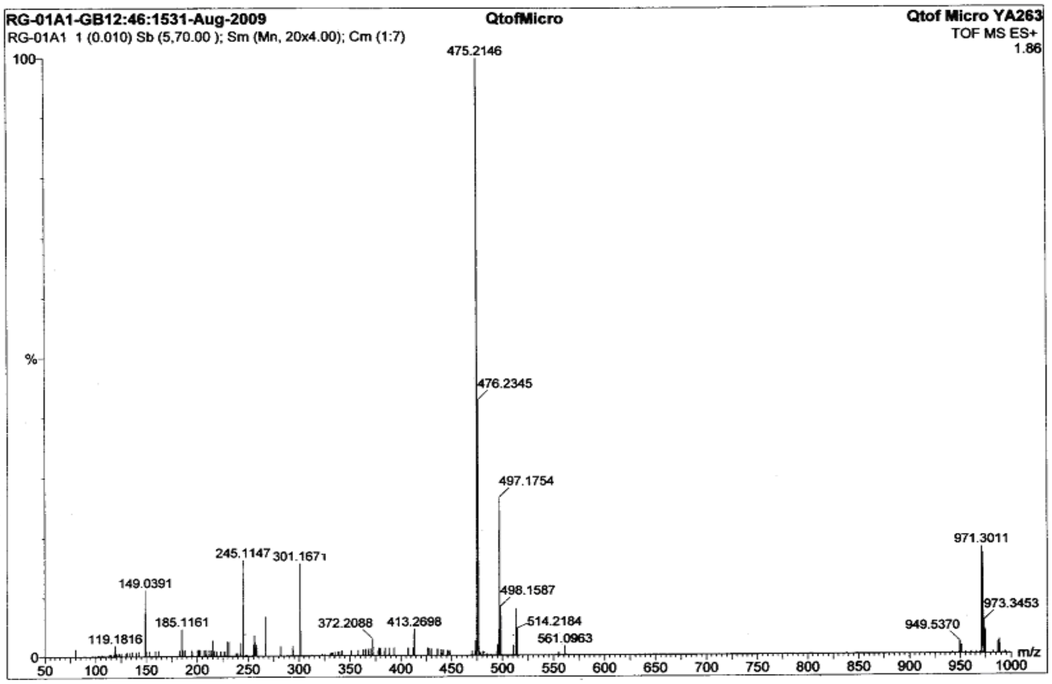

Figure 2: HRMS of receptor **1.**

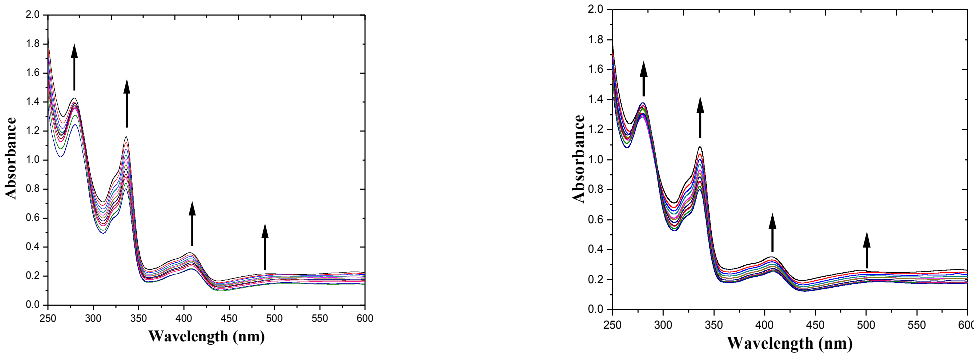

Figure 3: UV spectral change of receptor **1**(c = 1.1 x 10^{-4} M) dissolved in CH_3CN/H_2O (4:1 v/v) upon gradual addition of Cl^- (c = 7.196 x 10^{-4} M) (left side) and Br^- (c = 8.685 x 10^{-4} M) (right side).

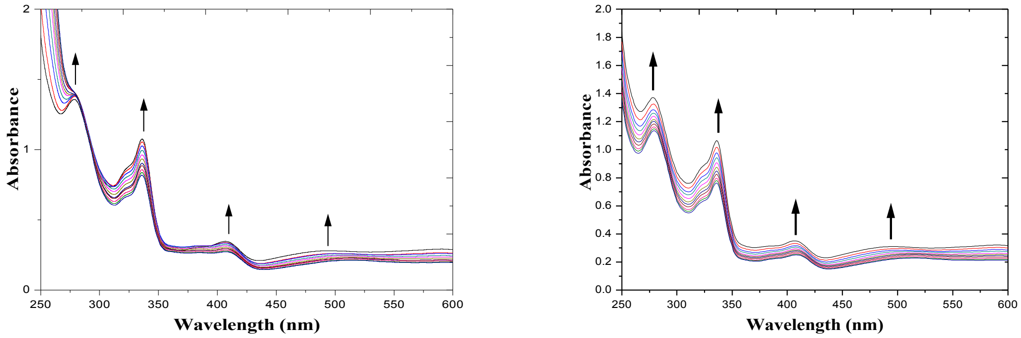

Figure 4: UV spectral change of receptor **1**(c = 1.1 x 10^{-4} M) dissolved in CH_3CN/H_2O (4:1 v/v) upon gradual addition of I^- (c = 7.85 x 10^{-4} M) (left side) and HSO_4^-. (c = 4.819 x 10^{-4} M) (right side).

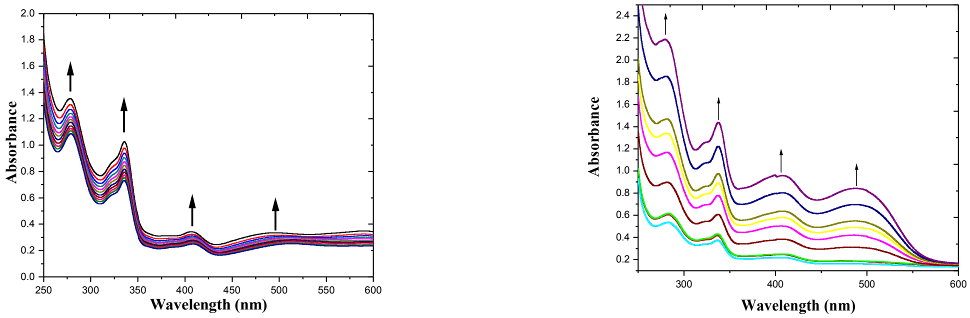

Figure 5: UV spectral change of receptor **1**(*c* = 1.1 x 10^{-4} M) dissolved in CH_3CN/H_2O (4:1 v/v) upon gradual addition of $H_2PO_4^-$ (*c* = 4.819 x 10^{-4} M) (left side) and UV spectral change of receptor **1**(*c* = 5.462 x 10^{-4}M) in DMSO upon gradual addition of F^- (*c* = 7.253 x 10^{-3} M) (right side) in DMSO.

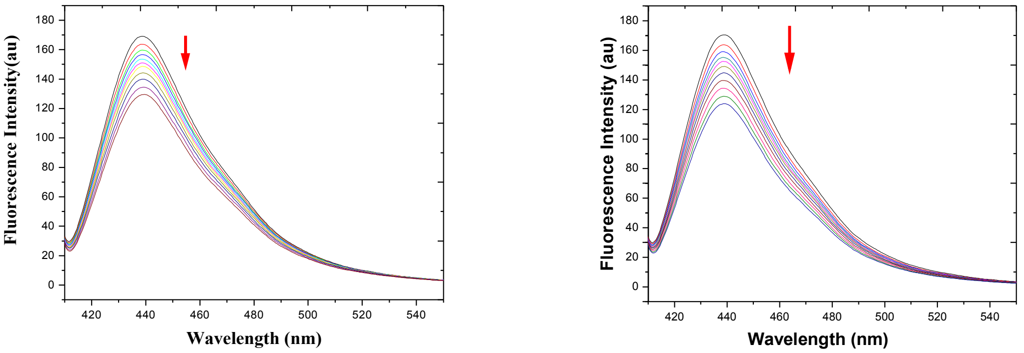

Figure 6: Fluorescence change of receptor **1** (*c* = 4.475 x 10^{-5} M) dissolved in CH_3CN/H_2O (4:1 v/v) upon gradual addition of Cl^- (*c* = 7.196 x 10^{-4} M) (left side) and Br^- (*c* =8.685 x 10^{-4} M) (right side).

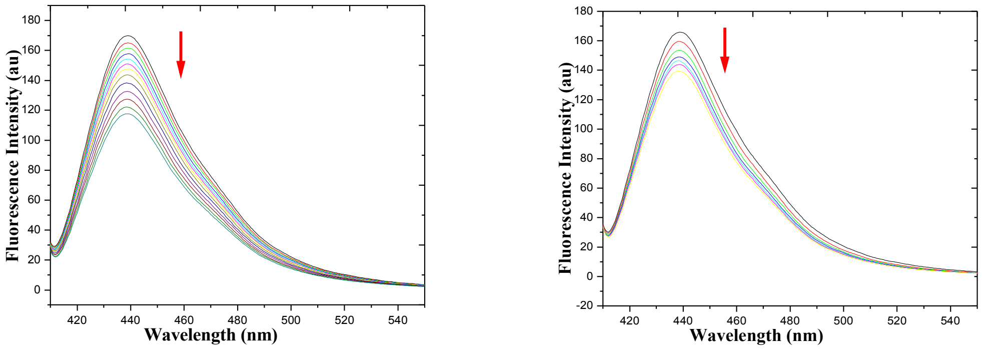

Figure 7: Fluorescence change of receptor **1** (c = 4.475 x 10^{-5} M) dissolved in CH_3CN/H_2O (4:1 v/v) upon gradual addition of I^- (c = 7.85 x 10^{-4} M) (left side) and HSO_4^- (c = 4.819 x 10^{-4} M) (right side).

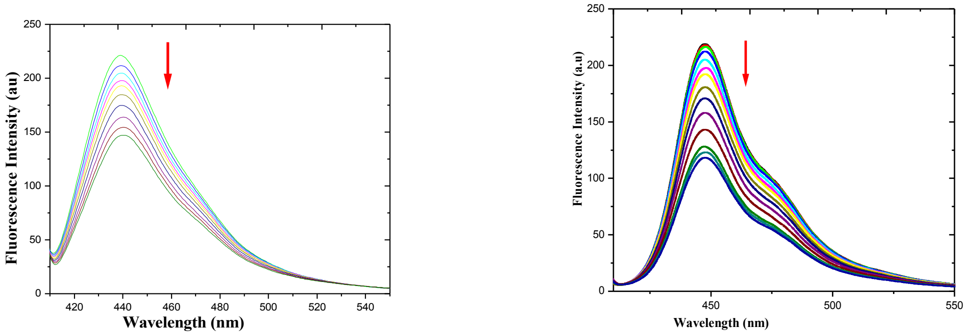

Figure 8: Fluorescence change of receptor **1** (c = 4.475 x 10^{-5} M) dissolved in CH_3CN/H_2O (4:1 v/v) upon gradual addition of $H_2PO_4^-$ (c = 8.2484 x 10^{-4} M) (left side) and fluorescence change of receptor **1** (c = 1.135 x 10^{-5} M) upon gradual addition of F^- (c = 6.351 x 10^{-5} M) (right side) in DMSO.
